# Pregnancy recognition trajectories: a needed framework

**DOI:** 10.1080/26410397.2023.2167552

**Published:** 2023-02-17

**Authors:** Joe Strong, Ernestina Coast, Emily Freeman, Ann M. Moore, Alison H. Norris, Onikepe Owolabi, Corinne H. Rocca

**Affiliations:** a PhD Candidate, London School of Economics and Political Science, London, UK. *Correspondence*: j.strong3@lse.ac.uk; b Professor of Health and International Development, London School of Economics and Political Science, London, UK; c Assistant Professorial Research Fellow, London School of Economics and Political Science, London, UK; d Principal Research Scientist, Guttmacher Institute, New York, NY, USA; e Associate Professor, College of Public Health, Ohio State University, Columbus, OH, USA; f Senior Global Director, MNCH/FP, IntraHealth International, Chapel Hill, NC, USA; g Professor, Department of Obstetrics, Gynecology and Reproductive Sciences, Advancing New Standards in Reproductive Health (ANSIRH), University of California San Francisco, Oakland, CA, USA

**Keywords:** pregnancy, pregnancy recognition, pregnancy testing, abortion, miscarriage

Pregnancy-related health care, including abortion care, is critically time bound. For pregnancies that will end in a birth, timely pregnancy recognition allows a person to access the recommended prenatal care, as well as make preparations for birth.^1^ For abortion-related care, the method, cost, and accessibility of care are all time-sensitive. Timely recognition of pregnancy is thus important for people to be able to make choices about their pregnancies and health care.^2^ Yet, little is known about how people recognise their pregnancies and how recognition impacts their subsequent pregnancy decision-making, care-seeking, and outcomes. There is no unifying framework to conceptualise how pregnancy recognition begins and unfolds.

Within our own work on abortion, we are aware of the role of pregnancy recognition in shaping the timing and accessibility of abortion care. Abortion-related care trajectories are sensitive to any potential delays in care-seeking.^3^ Most legal frameworks impose restrictions on abortions, and many include gestational limits beyond which a person cannot obtain care (except under specific exemptions). Despite recent progress in abortion legislation in countries such as Ireland and Argentina, some US states have lowered the legality of care to before 6 weeks gestation, necessitating very early pregnancy recognition. Such limits on abortion remove the possibility of time for changing or evolving decision-making about a pregnancy.^4^

The research language around this concept is varied, including pregnancy suspicion, awareness, testing, and confirmation.^5,6^ Suspicion and awareness of a pregnancy can refer to the earliest signs, symptoms, or indications of a pregnancy; they may also stem from the nature of the sexual encounter and the non-/use of contraceptives. Confirmation might include testing but could consist of any number of factors (e.g. symptoms, bodily changes) that provide a person with some evidence of a pregnancy. Increasingly, clinical recommendations link confirmation to medical tests, including at-home urine tests.

In this commentary, we bring together these linked but separate components under the term “pregnancy recognition trajectory” to describe the varied processes by which people come to know they are pregnant. We demonstrate that people’s pregnancy recognition trajectories can be non-linear, complex, and shaped by individual, interpersonal, community, and structural factors. Building on the abortion-related care trajectory developed by Coast, Norris et al.,^3^ we use “trajectory” to incorporate time within the process of recognising a pregnancy. The aims of this commentary are twofold: to demonstrate the need for a comprehensive pregnancy recognition trajectory framework to guide future research; and to examine the critical components of these trajectories. This commentary encourages the development of future data collection on pregnancy recognition in more meaningful ways**.**

## The contours of a trajectory

Pregnancy recognition can be non-linear over time, necessitating an understanding of how to represent and understand a trajectory as a composite of events and processes with varying speeds and directions*.* Pregnancy recognition is subjective and can be shaped by normative ideas of what constitutes a pregnancy. Sensitive to these subjectivities, there is no universal “start” or “end” point of a pregnancy recognition trajectory. In particular, pregnancies can include liminal stages, in which a person might not consider themselves fully or definitively pregnant*,* instead operationalising the cultural and contextual uses of “productive” pregnancy ambiguity.^7^

While a starting point could be a sexual encounter, a person’s intentions, desires, and fertility treatment can all shape when a trajectory begins. Moreover, recognition is facilitated by increased awareness of the likelihood of a pregnancy, which includes knowledge of fecundability at the time of conception and the use and efficacy of different contraceptives. For example, believing that a contraceptive has failed might be the “start” of a pregnancy recognition trajectory for someone who knows there is an increased likelihood of having become pregnant, while a lower frequency of sex without contraception can lead to lower perceptions of risk of pregnancy.^8^ In contrast, a person’s perception that they are infertile or unable to conceive could mean that they “start” their pregnancy recognition trajectory later.^8^ The endpoint of a trajectory could be a live birth, stillbirth, miscarriage, or abortion. For example, a person might not experience bodily changes during a pregnancy and not recognise they were pregnant until they experience a miscarriage or live birth.

Demographic differences in pregnancy recognition trajectories offer insight into individual, community, and structural influences. The age of a person shapes their belief that signs or symptoms are linked to pregnancy - particularly if they consider themselves too young or old to be pregnant.^9^ Whether a person has experienced pregnancy before influences their ability to recognise one, using their lived experience, whilst it might also reduce the likelihood of recognising a pregnancy if the signs and symptoms are different from prior pregnancies.^9^ Experiences of violence and the nature of the sexual encounter could compound emotional and physical barriers to recognition, making critical the consideration of the relationship between trauma and recognition.

Pregnancy recognition requires knowledge of and information on sexual and reproductive health, including an understanding of how pregnancies occur. For people seeking testing for a pregnancy, knowledge of how and where to obtain different tests (at-home or facility urine tests, ultrasounds, etc.) is fundamental. Knowledge of the potential signs and symptoms of pregnancy is another critical component. Late menstrual periods are a commonly recognised sign of pregnancy.^6^ However, many people have irregular periods, recent menarche, or continued bleeding during pregnancies. Variation in these physiological experiences affects the degree to which menstrual changes serve as a cue to pregnancy recognition.^10^

Knowledge intersects with personal experiences, impacting the importance a person places on a sign or symptom. Bodily changes, such as nausea or vomiting, might be recognised as pregnancy symptoms if a person has been pregnant before, believes pregnancy is likely, or in contexts where these are commonly recognised signs of pregnancy. Symptoms may be initially linked to illnesses with similar symptoms, before being connected to a pregnancy. Grappling with knowledge and information, therefore, requires locating trajectories at the individual, community, and structural levels.

The perception that physiological signs and symptoms could be associated with a pregnancy highlights the important intersections between knowledge and psychological factors. A person’s belief that they could be pregnant is shaped by numerous factors, which might include desires to avoid being confronted by the reality of recognising a pregnancy. This belief can also be shaped by the fear of a pregnancy and its social repercussions, or the desire to avoid recognising a pregnancy too early in case of early miscarriage. By contrast, psychological factors might mean that people begin a pregnancy recognition trajectory while not being pregnant. This includes individuals who desire to be pregnant and might interpret signs and symptoms to be linked to a (non-existent) pregnancy, as well as “pregnancy scares” for those who do not desire to be pregnant but have heightened concern they are at risk.

Pregnancy testing is a core means by which to confirm a pregnancy. A person needs information about whether, how and where they can obtain testing, financial resources to pay for a health visit or test, and capacity to reach a pharmacy, clinic, or the internet. In addition to overcoming these obstacles, some individuals are less trusting of the accuracy of tests, and others may avoid tests to preclude having “proof” of pregnancy. (Ralph 2022)

There are also situations in which a person might not wish to know they are pregnant. In contexts where menstrual regulation services are offered (e.g. Bangladesh), a positive pregnancy test might cause someone to believe they cannot (or that they should not) access these services. In Bangladesh, where abortion is criminalised, confirming a pregnancy can legally preclude someone from accessing menstrual regulation services, which are offered up to 10 weeks after the last menstruation. In addition, an individual might seek to not fully recognise a pregnancy before taking medical abortion pills as a means of navigating perceptions that abortions are “unacceptable”. Evidence indicates that US women are interested in “missed period pills” – mifepristone and/or misoprostol – as a way of ensuring they are not pregnant without pregnancy testing.^11^ Menstrual regulation and missed period pills illustrate the necessity of understanding situations and conditions in which pregnancy recognition is deliberately avoided, and the importance of this within the trajectory framework.

Pregnancy recognition trajectories are tethered to pregnancy disclosure. Decisions of whether, when, and to whom to disclose a pregnancy suspicion or confirmation can be shaped by notions of safety, secrecy, and necessity where abortions are facilitated through a *constellation of actors* (e.g. friends, partners, activists, providers, etc.)*,* who can provide assistance when accessing care.^12^ Individuals might involve another person in helping them recognise their pregnancy, by discussing signs and symptoms, or in seeking emotional, financial, or other support in accessing medical testing. Medical testing, in turn, might involve having to disclose a suspected pregnancy to a pharmacist or other provider. Inability to recognise a pregnancy could also lead to other people noticing first - for example, observing bodily changes. Understanding pregnancy recognition trajectories is, therefore, tied to understanding the ability for a person to retain secrecy, privacy, bodily autonomy, and choice in their subsequent pregnancy-related decision-making.^13^

Existing modes of discrimination and oppression intersect and amplify factors that shape pregnancy recognition trajectories. Economic structures – classed, racialised, aged, abled, gendered – that marginalise key groups can contribute to difficulties accessing medical pregnancy testing. Social structures can determine whether stigma and judgement delay or block the ability to seek information and care – for example, for an unmarried person in a context where pregnancy outside of marriage is stigmatised, or for a person with disabilities in a context where disabled people are discriminated against for being pregnant. Educational and information structures that are designed in exclusionary ways can similarly impact disabled people, including reducing the accessibility of places to procure medical tests or find information on signs and symptoms of pregnancies. Evidence suggests that perceptions of fecundity are racialised, intersecting with inequalities as well as prior fertility.^14^ Moreover, the motivations to recognise a pregnancy can be shaped by a person’s experiences and desired pregnancy recognition trajectories should not be assumed as the same across populations.

## Implications

Pregnancy recognition trajectories matter. The inability to recognise a pregnancy can compromise the choice and autonomy of a person to make decisions about their pregnancy and related health care, contributing to continued reproductive injustice and inequities. Yet, the factors outlined in this commentary, which shape pregnancy recognition, are myriad, complex, non-linear, and sparsely evidenced. Incorporating questions – quantitative and qualitative – that relate to pregnancy recognition in future research agendas will generate more nuanced evidence to help understand and interrogate pregnancy experiences and their outcomes. This evidence will further develop our understanding of pregnancy recognition trajectories and their outcomes in our field.

While the pregnancy recognition trajectory is important for all types of pregnancy and pregnancy outcomes, it is particularly important for abortion. Obstacles to abortion care intersect with gestational timelines, meaning that the timing of pregnancy recognition can be a critical determinant of the availability, accessibility, affordability, and legality of care.^15^ Our aim is to encourage future research on pregnancy recognition trajectories across different contexts and populations, with the goal of interrogating how we understand and measure pregnancy recognition. As future research investigates pregnancy recognition trajectories, the development of a novel framework will facilitate in-depth analysis. New evidence, in turn, can iterate this framework and allow for the generation of more effective policies and programmes for helping people recognise a pregnancy at the time and in the way they desire, if they desire.

## References

[1] World Health Organization. WHO recommendations on health promotion interventions for maternal and newborn health. Geneva: World Health Organisation; 2015. 26180864

[2] World Health Organization. Abortion care guideline. Geneva: World Health Organization; 2022.

[3] Coast E, Norris AH, Moore AM, et al. Trajectories of women's abortion-related care: a conceptual framework. Soc Sci Med. 2018;200:199–210. 29421467 10.1016/j.socscimed.2018.01.035

[4] Macleod CI. Public reproductive health and ‘unintended’ pregnancies: introducing the construct ‘supportability’. J Public Health. 2016;38(3):e384–e391. 10.1093/pubmed/fdv123 26354998

[5] Foster DG, Gould H, Biggs MA. Timing of pregnancy discovery among women seeking abortion. Contraception. 2021;104(6):642–647. 34363842 10.1016/j.contraception.2021.07.110

[6] Somefun OD, Harries J, Constant D. Reproductive awareness and recognition of unintended pregnancy: young women, key informants and health care providers perspectives in South Africa. Reprod Health. 2021;18(1):211. 34702283 10.1186/s12978-021-01262-0 PMC8549136

[7] Bell SO, Fissell ME. A little bit pregnant? Productive ambiguity and fertility research. Popul Dev Rev. 2021;47(2):505–526.

[8] Bell SO, Gemmill A. Perceived likelihood of becoming pregnant and contraceptive use: findings from population-based surveys in Côte d'Ivoire, Nigeria, and Rajasthan, India. Contraception. 2021;103(6):431–438. 33587907 10.1016/j.contraception.2021.02.002 PMC8129551

[9] Ralph LJ, Foster DG, Barar R, et al. Home pregnancy test use and timing of pregnancy confirmation among people seeking health care. Contraception. 2022;107:10–16. 34748750 10.1016/j.contraception.2021.10.006

[10] Nobles J, Cannon L, Wilcox AJ. Menstrual irregularity as a biological limit to early pregnancy awareness. Proc Natl Acad Sci USA. 2022;119(1):e2113762118. 34969843 10.1073/pnas.2113762118 PMC8740731

[11] Sheldon WR, Mary M, Harris L, et al. Exploring potential interest in missed period pills in two US states. Contraception. 2020;102(6):414–420. 32916168 10.1016/j.contraception.2020.08.014

[12] Berro Pizzarossa L, Nandagiri R. Self-managed abortion: a constellation of actors, a cacophony of laws? Sex Reprod Health Matters. 2021;29(1):1899764. 33764856 10.1080/26410397.2021.1899764 PMC8009018

[13] Nandagiri R. “Can you keep a secret?”: methodological considerations for qualitative abortion research. London: UCL Qualitative Health Research Network; 2020.

[14] Gemmill A. Perceived subfecundity and contraceptive use among young adult U.S. women. Perspect Sex Reprod Health. 2018;50(3):119–127. 29969174 10.1363/psrh.12072

[15] Watson K, Angelotta C. The frequency of pregnancy recognition across the gestational spectrum and its consequences in the United States. Perspect Sex Reprod Health. 2022;54(2):32–37. 35576053 10.1363/psrh.12192 PMC9321827

